# Supplementary material for: Patient-specific Alzheimer-like pathology in trisomy 21 cerebral organoids reveals BACE2 as a gene dose-sensitive AD suppressor in human brain
Source: Mol Psychiatry. 2020 Jul 10;26(10):5766–88. doi: 10.1038/s41380-020-0806-5 (PMC8190957; doi:10.1038/s41380-020-0806-5)
Supplement: Supplementary file 2 — Supplementary Figure Legends [file 41380_2020_806_MOESM2_ESM.docx]

**Supplementary Figures**

**Supplementary Fig. 1.** **Cerebral organoids express cortical neuronal layer-specific and astrocyte markers.** Representative images of isogenic D21 (**a-c**), isogenic T21 (**d-f**), and non-isogenic QM-DS1 (T21) (**g-i**), QM-DS2 (T21) **(j-l**) and QM-DupAPP (**m-o**) organoids are shown, confirming expression of TBR1 (layer IV), CTIP2 (layer V), FOXG1 (layers II and III), BRN2 (layer VI), REELIN (layer I), SATB2 (layer III), GFAP (astrocytes) and 3R-Tau (neurons). Final image in each row is a merge of individual antibodies in that row. Scale bar: 100μm.

**Supplementary Fig. 2. Comparison of the proportions of neurons and astrocytes to total cells** **in cerebral organoids**. Isogenic D21 and T21 cerebral organoids, as well as organoids from DupAPP, QM-DS1 and QM-DS2 iPSCs generated mostly neurons and a small proportion of astrocytes, with none or minor differences in the proportion of astrocytes or neurons between the lines. Quantification was performed on 8 representative (Z-stack) figures each from three individual organoids per genotype. Neurons are labelled with MAP2 and astrocytes with GFAP, each marker was normalized to DAPI. Image analysis was performed using IMARIS software. Error bars: standard error. Scale bar: 20 μm.

**Supplementary Fig. 3. SNP arrays confirmed trisomy of chromosome 21 in all the iPSC lines used in this study, or in the case of QM-DupAPP, the partial duplication of a 580kbp segment of chromosome 21.** The duplicated region in QM-DupAPP is also shown in a magnified image. Representative images of QM-DS1 and QM-DS2 confirm the expression of pluripotency markers (SSEA-4, Oct4 and Tra 1-60), scale bar: 300μm, and Alkaline Phosphatase activity, scale bar: 100μm.

**Supplementary Fig. 4. Quantitative comparison of 100DIV isogenic T21 and D21 organoid “cortical” regions by FISH and I.F. a** For Fluorescence In Situ Hybridisation (FISH), organoid slices were processed and probed with a MetaSystems Probes for chromosome 21 (red) and chromosome 13 (green). Quantification was performed automatically using IMARIS software (nuclei were scored for 1, 2, 3 or >3 spots n>500 nuclei for each line). Scale bar: 3μm. **b** APP-4G8 antibody (green), BACE2 (red), MAP2 (magenta), DAPI (blue). Scale bar: 10μm. **c** Aβx-34 neo-epitope specific antibody (green), BACE2 (red), MAP2 (magenta), DAPI (blue). Scale bar: 10μm. **d** Quantification was performed blinded to the genotype, on 5 independent images representing three individual organoids per genotype, and containing 3,000-4,000 cells per image. Only images within the “cortical” part of the organoid were considered for the analysis. Graphs show total fluorescence intensity of positive signals for each wavelength for a given antibody, normalised by the total fluorescence intensity of MAP2 as a pan-neuronal marker. Additional staining (not shown) was analysed for Aβx-40 neo-epitope specific antibody. Image analysis was performed using IMARIS software. Error bars: standard error, and p-values: calculated after Holm-Bonferroni correction (α=0.05) of sequential two-tailed student t-test comparisons.

**Supplementary Fig. 5. Simplified schematic representation of APP695 from amino acid 500 to the C-terminal.** The amino acid cleavage site for each secretase is noted in parentheses, and for sites in Aβ the respective peptide position is also noted. Secretases for each cleavage site are η: MT5-MMP, δ: AEP, β: BACE1, α: ADAM10, θ: BACE2, γ: gamma secretase complex. In addition to it’s role as a θ-secretase, in which C99 is cleaved to preclude Aβ formation, in this report we highlight the role of BACE2 to act on Aβ as substrate, as an Aβ-degrading protease (AβDP).

**Supplementary Fig. 6. Conditioned media from isogenic D21 and T21 organoids was compared by IP-MS and ELISA. a** IP-MS showed that all amyloid peptide species were increased in T21 compared to D21 samples. **b** Despite the increase in peptides from T21 organoids, no significant change in ratio of Aβ40:Aβ42 was detected by ELISA **c** The increase in peptide concentrations for Aβ38, Aβ40 and Aβ42 in T21 organoid CM was confirmed by MSD ELISA. **d** representative Aβ IP-MS spectra of organoid CM (*co-IP-ed unknown non-β-amyloid peptide). **e** Areas under the peak (used as a readout in all IP-MS experiments) show near linear correlation with ELISA-measured levels of Aβ peptides.

**Supplementary Fig. 7. Aβx-34 colocalises with BACE2 much more than with BACE1 in T21 cerebral organoids.** **a** Pairwise Pearson’s coefficient of colocalised volume for a pair of co-stained antibodies: Aβx-34 in all combinations, either BACE1 or BACE2 as a second antibody, and a marker of the sub-cellular vesicle compartment (shown at the bottom of each 3-columns histogram) as a third antibody. Error bars: standard error, and p-values: standard one-way ANOVA using post-hoc Bonferroni correction for multiple comparisons calculation. Representative individual z-slice images for the calculations performed in a are shown in **b** and c. b BACE1 (green), **c** BACE2 (green), with either Aβx-34, Sortilin or LAMP2 (red) and DAPI (blue). Scale bar: 10μm.

**Supplementary Fig. 8. Validation and controls for immunohistochemistry. a** Sudan black B was used to confirm the specificity of the Aβx-34 and BACE2 antibodies in human brain sections, and to eliminate lipofuscin autofluorescence. Three different human brain samples were used: DS-AD1, DS (28 yrs) pre-AD and euploid sporadic AD (73 yrs). Both antibodies show the same pattern of expression and colocalisation after Sudan black B staining (white arrows: intraneuronal fine-vesicular pattern and black arrows with white arrowhead: amorphous extra-cellular aggregates) except for a loss of the large intraneuronal spherical granules (white arrowheads, Fig. 3), which are likely lipofuscin. Scale bar: 5μm. **b** and **c** Chromogenic, immunohistochemical analysis of the human brain sections of DS-AD1, stained using polymer-HRP/AP double-staining kit. **b** The primary antibody against BACE2 was labelled with DAB (brown) and primary antibody against Aβx-34 neo-epitope was labelled with GBI-permanent-red (red); **b(i)** is a zoomed-in inset of the rectangle in B. c same as b, but both antibodies were pre-absorbed for 12 hours, and incubated overnight, with the excess of immunogenic peptide for the BACE2 antibody; **c(i)** is a zoomed-in inset of the rectangle in c. d and e: BACE2 antibody specificity control for immunofluorescence on T21 organoids (100DIV). Scale bar: 10μm. **d** Immunofluorescent staining with Aβx-34 and BACE2, **e** same as **b** and **d**, but both antibodies were pre-absorbed for 12 hours, and incubated overnight, with the excess of immunogenic peptide for the BACE2 antibody. The specificity for the neo-epitope specific antibody against Aβx-34 was extensively proven in a previous report (Cabrera et al., 2018). **f** and **g** In order to distinguish the contribution of lipofuscin auto-fluorescence to the colocalised signals, specificity of primary antibodies (Aβx-34 and BACE2) has been validated using Lambda (λ) scan function on confocal microscope (see Methods). **f** Aβx-34 shows specific peak in different ROI and uniform pattern on the three different human brain samples: DS-AD1 (59 yrs), DS (28 yrs) pre-AD and DS (8 months). As negative control of staining, DAPI and secondary antibody alone were used. **g** BACE2 also shows specific peak in different ROI and uniform pattern in human brain. **h** secondary antibody alone control. Scale bar: 20μm.

**Supplementary Fig. 9. Validation of the genome integrity of CRISPR-edited iPSCs by SNP array and paralogous amplification quantitative pyrosequencing, and that CRISPR-edited cerebral organoids express cortical neuronal layer-specific markers.**

**a** Following CRISPR editing, C5∆7 iPSC line was assessed by SNP array and no genomic alternations were detected compared to the parental C5 iPSC line. B-allele frequency and LogR ratio plots comparing these two lines are shown for chromosome 21. Data for the whole genome available on request. **b** To further confirm retention of trisomy after BACE2 CRISPR editing, selected other genes on chromosome 21 were validated as trisomic using quantitative paralogous amplification/pyrosequencing method. GABPA and ITSN allele number were quantified relative to paralogous sequence mismatches on other chromosomes. GABPA and ITSN both show approximate 60:40 ratios for trisomic cells, and 50:50 ratios for disomic cells as expected. Quantified nucleotides are shown on the pyrogram in shaded grey boxes and the relative values for the corresponding peaks are shown. **c** Representative images of T21C5Δ7 organoids at DIV48 are shown, confirming expression of REELIN (layer I), BRN2 (layer VI), TBR1 (layer IV), FOXG1 (layers II and III), CTIP2 (layer V), SATB2 (layer III) and pan-neuronal makers MAP2 and 3R-Tau. Final image in each row is a merge of individual antibodies in that row. Scale bar: 50μm.

**Supplementary Fig. 10. CRISPR/SpCas9-HF1-mediated reduction of BACE2 copy number from 3 to 2 in the T21C5 hiPSC line, reduced BACE2 protein expression to disomic levels, but does not alter the level of APP protein.** Western blot stained with anti-BACE2 antibody or anti-APP antibody of the lysates of the iPSC line Δ7 compared to the wt T21C5, and D21C3 iPSC lines. Quantification of the total actin-normalised BACE2 signal showed a 27% reduction in Δ7 compared to T21 unedited line, and no significant difference compared to D21 control. Quantification of the total actin-normalised APP signal showed no significant difference between Δ7 and unedited T21 line, whereas they both had significantly higher APP protein levels compared to the disomic control line. Error bars: standard error, p-values after standard one way ANOVA and Tukey’s multiple comparisons test.

**Supplementary Fig. 11. Staining of extracellular β-amyloid deposits in organoids with two different methods. a** Early AD-like pathology was provoked in the Δ7 organoids. Staining with amyloid specific dye (AmyloGlo) and nuclear dye (DRAQ5). Top row: wt unedited control T21C5 (100DIV), Middle row: T21C5Δ7 (48DIV) and bottom row: T21C5Δ7 (96DIV); amyloid deposits are seen in Δ7 after 48DIV, but not its parental clone, at same organoid age. **b** Staining with Thioflavine S shows the same plaque-like pathology observed using AmyloGlo in the Δ7 after 48DIV. Scale bar: 20μm.

**Supplementary Fig. 12. Cell death and neuronal loss in CRISPR-edited T21C5Δ7 organoids.** Quantification was performed on 8 representative (Z-stack) figures from three individual organoid per genotype. Number of DAPI+ nuclei are shown in the volume of 10 000 µm^3^. Graph show decreased number of nuclei in CRISPR-edited T21C5Δ7 (DIV48) organoids compared to parental T21C5 organoids and significantly decreased number of nuclei in 96DIV organoids (p<0.0001). Significantly decreased number of nuclei were observed between DIV48 and DIV96 in CRISPR-edited T21C5Δ7 (p<0.001). Error bars: standard error, and p-values after standard one-way ANOVA and Tukey’s multiple comparison test.

**Supplementary Fig. 13. Electron micrographs of negatively stained filaments isolated from insoluble fraction of the AD-like pathology containing organoid lysates. a, b** representative straight filaments found in the lysates from the organoids T21C5Δ7 and QM-DupAPP, respectively. **c** Aβ1-40 synthetic peptide fibrils grown in vitro. Scale bars: a, c: 20nm, b: 40nm.

**Supplementary Fig. 14. shRNA-mediated knockdown of BACE2 in QM-DS6 reduced protein expression in iPSCs and provoked AD-like pathology.** **a** Stable iPSCs lines were generated expressing shRNA against BACE2 or a control scrambled (SCR) shRNA. Reduction of BACE2 expression compared to the scrambled control was confirmed by Western blotting, n=4, p=0.04, Student’s t-test. **b-d** Cerebral organoids were generated from stable shRNA-expressing iPSCs and analysed after 46DIV. **b** the reduction in BACE2 expression resulted in the appearance of AD-like pathology (AmyloGlo deposits), scale bar: 50μm. **c-d** BACE2 shRNA also resulted in a significant increase in the number of neurons expressing conformationally altered Tau (TG3+). Scale bar: 20μm. Three different organoids and a minimum of 10 images per condition were used for analysis, p=0.0025, two-tailed Student’s t-test. Error bars: standard error.

**Supplementary Fig. 15. Secondary antibody alone controls for organoid immunostaining.** DAPI staining confirms the presence of cells, but no unspecific signal from secondary antibodies. Scale bar: 20μm.
