# Supplementary figures and images for: Patient-specific Alzheimer-like pathology in trisomy 21 cerebral organoids reveals BACE2 as a gene dose-sensitive AD suppressor in human brain
Source: Mol Psychiatry. 2020 Jul 10;26(10):5766–88. doi: 10.1038/s41380-020-0806-5 (PMC8190957; doi:10.1038/s41380-020-0806-5)

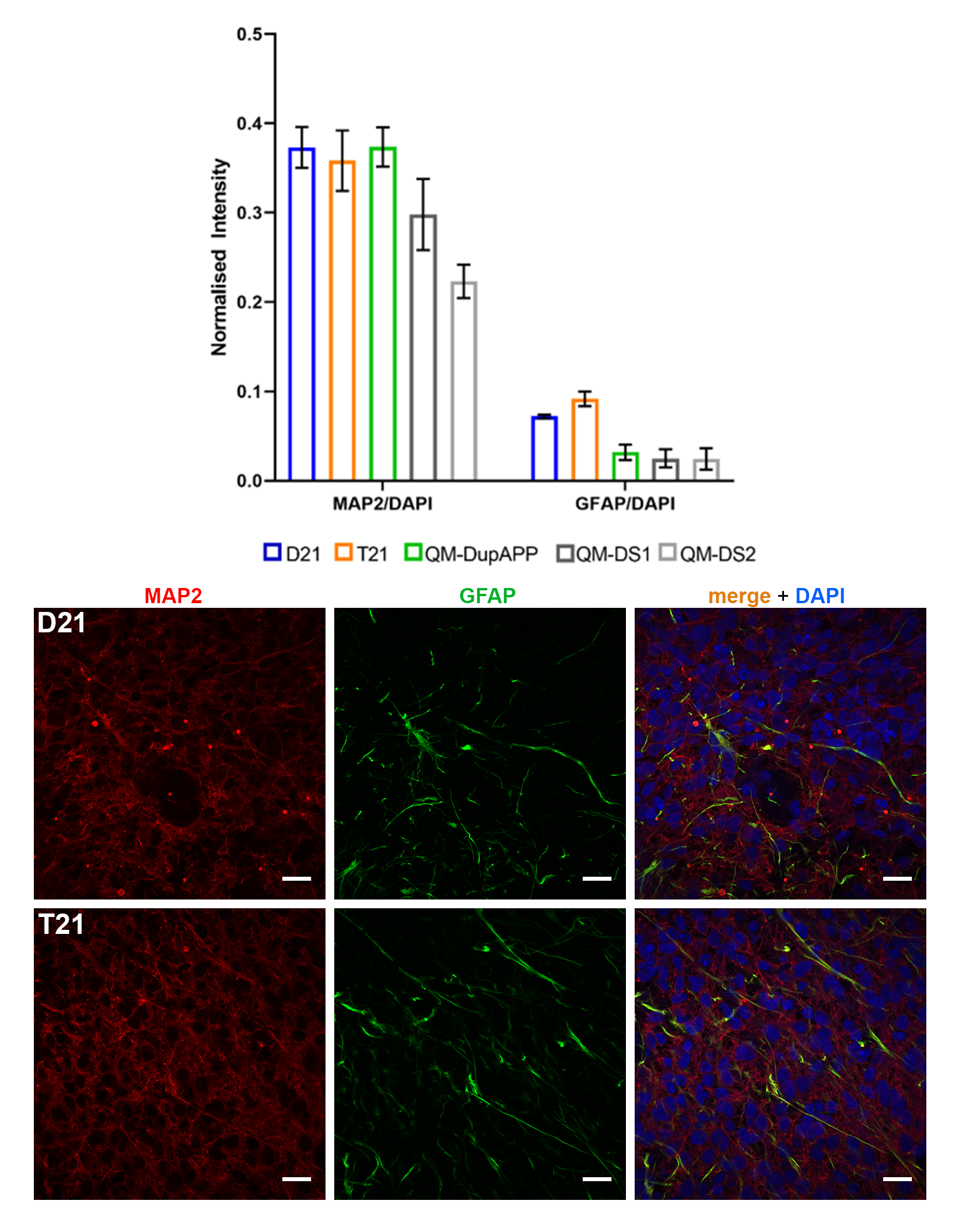

Supplement: Supplementary file 4 — Supplementary Figure 2 [file 41380_2020_806_MOESM4_ESM.tif]

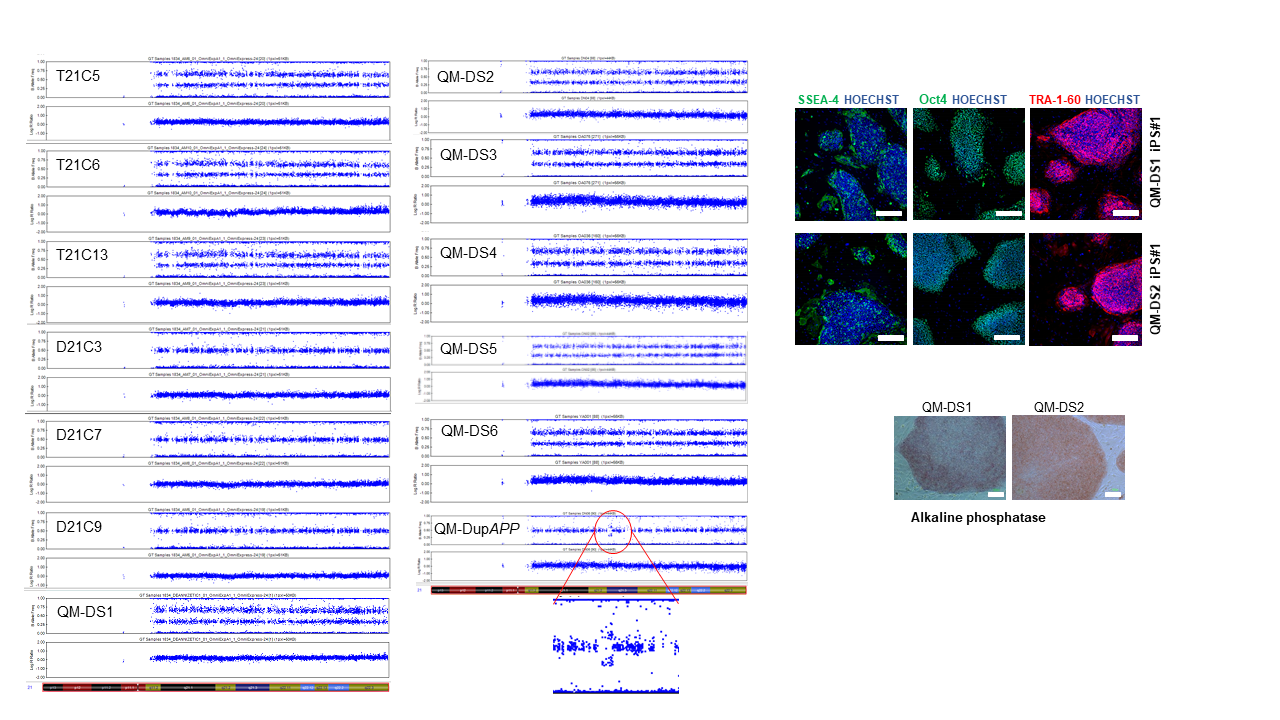

Supplement: Supplementary file 5 — Supplementary Figure 3 [file 41380_2020_806_MOESM5_ESM.tif]

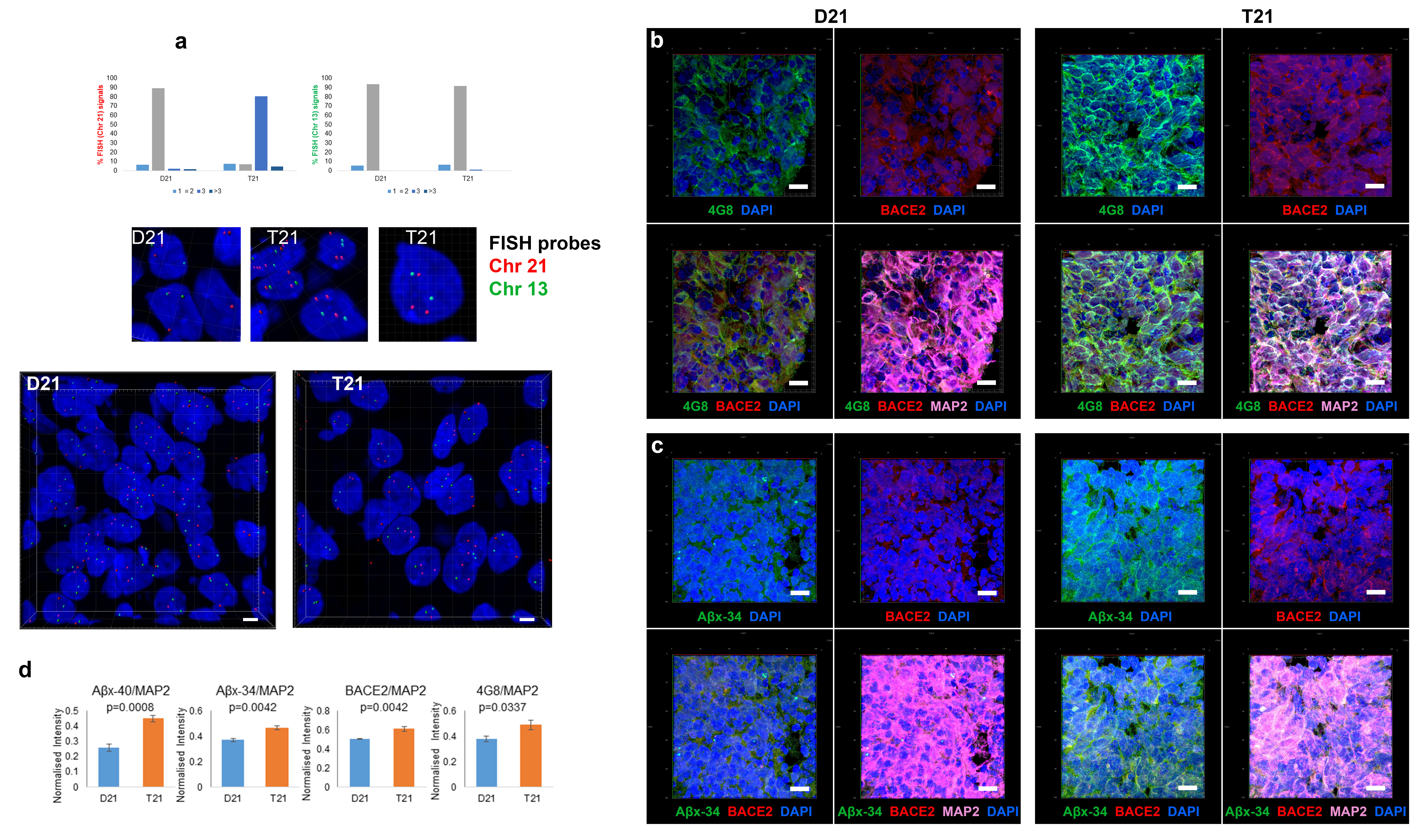

Supplement: Supplementary file 6 — Supplementary Figure 4 [file 41380_2020_806_MOESM6_ESM.tif]

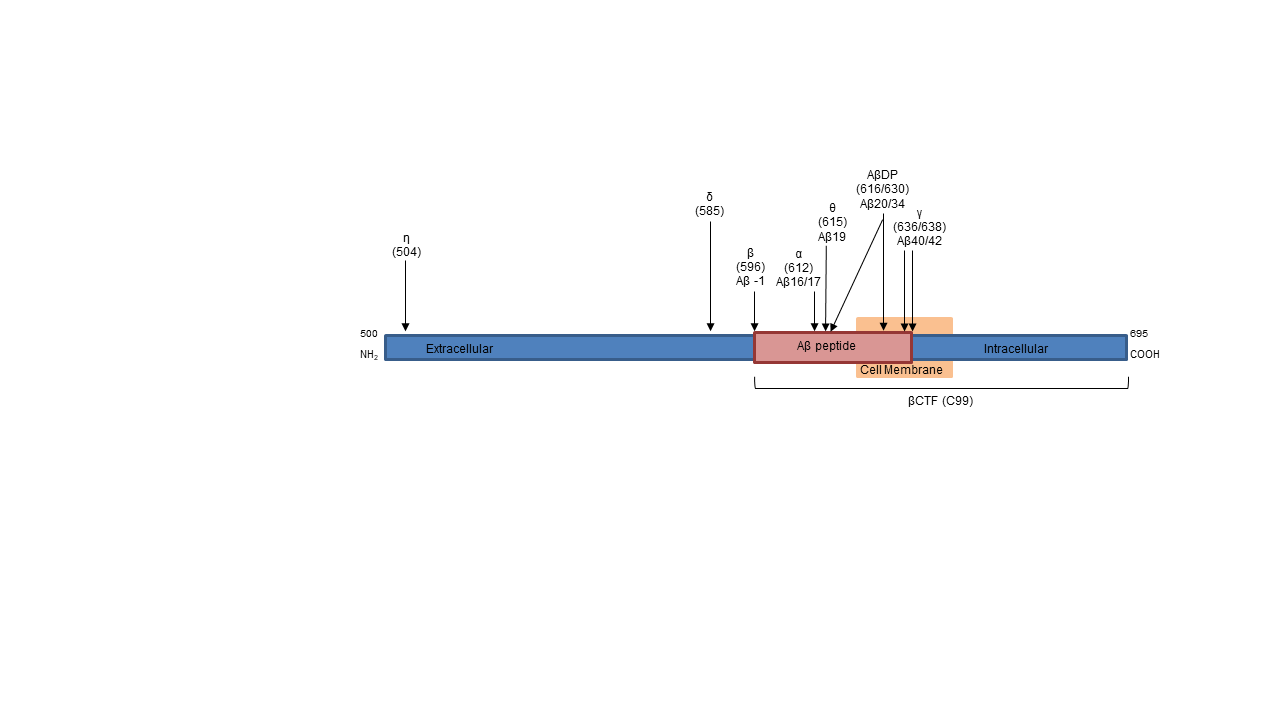

Supplement: Supplementary file 7 — Supplementary Figure 5 [file 41380_2020_806_MOESM7_ESM.tif]

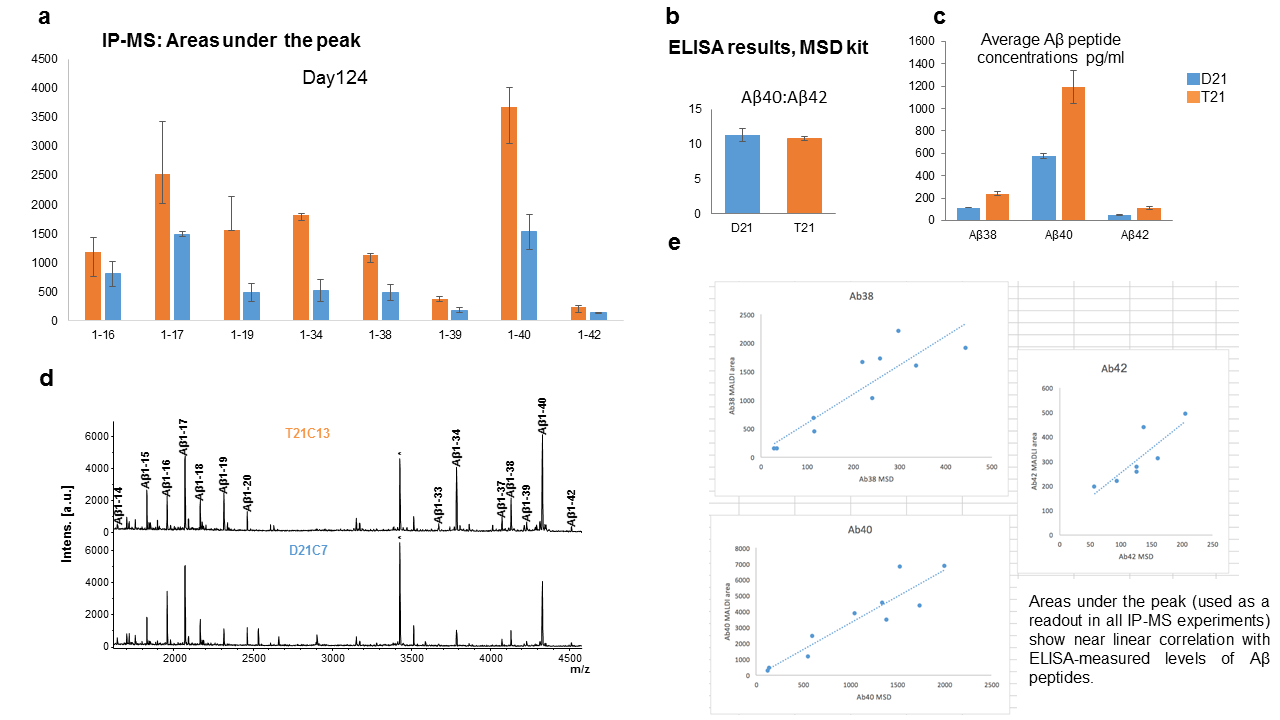

Supplement: Supplementary file 8 — Supplementary Figure 6 [file 41380_2020_806_MOESM8_ESM.tif]

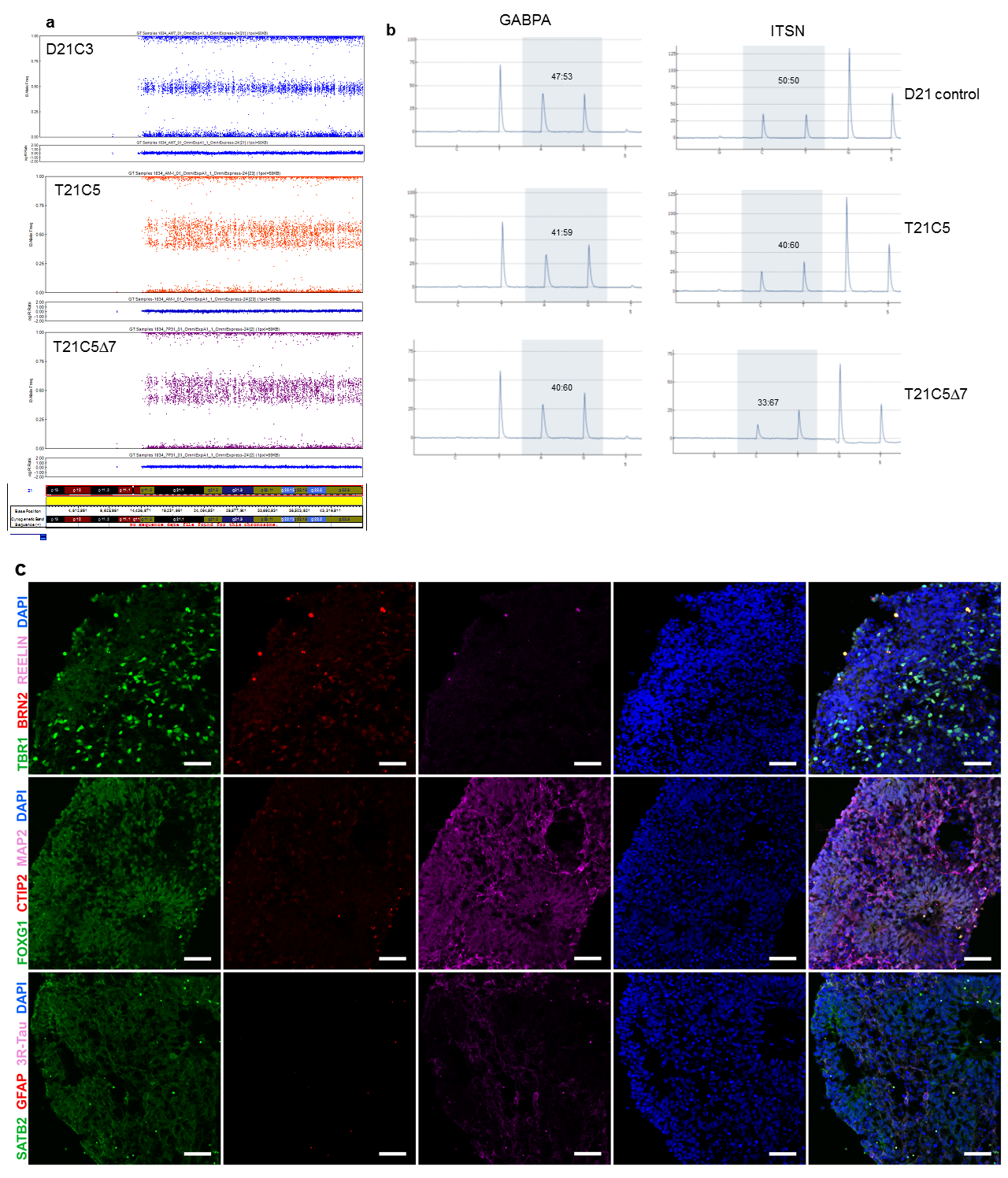

Supplement: Supplementary file 11 — Supplementary Figure 9 [file 41380_2020_806_MOESM11_ESM.tif]

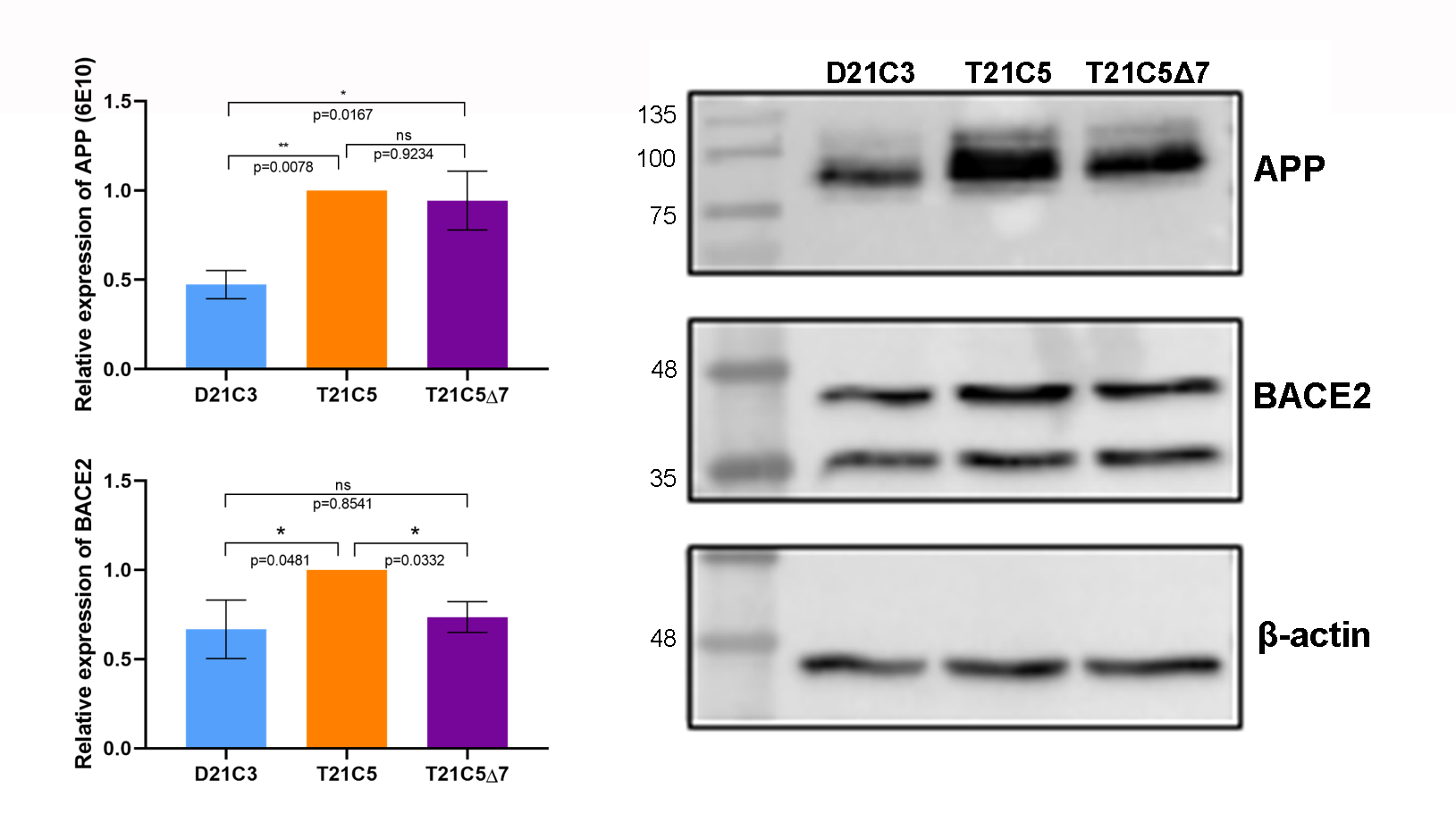

Supplement: Supplementary file 12 — Supplementary Figure 10 [file 41380_2020_806_MOESM12_ESM.tif]

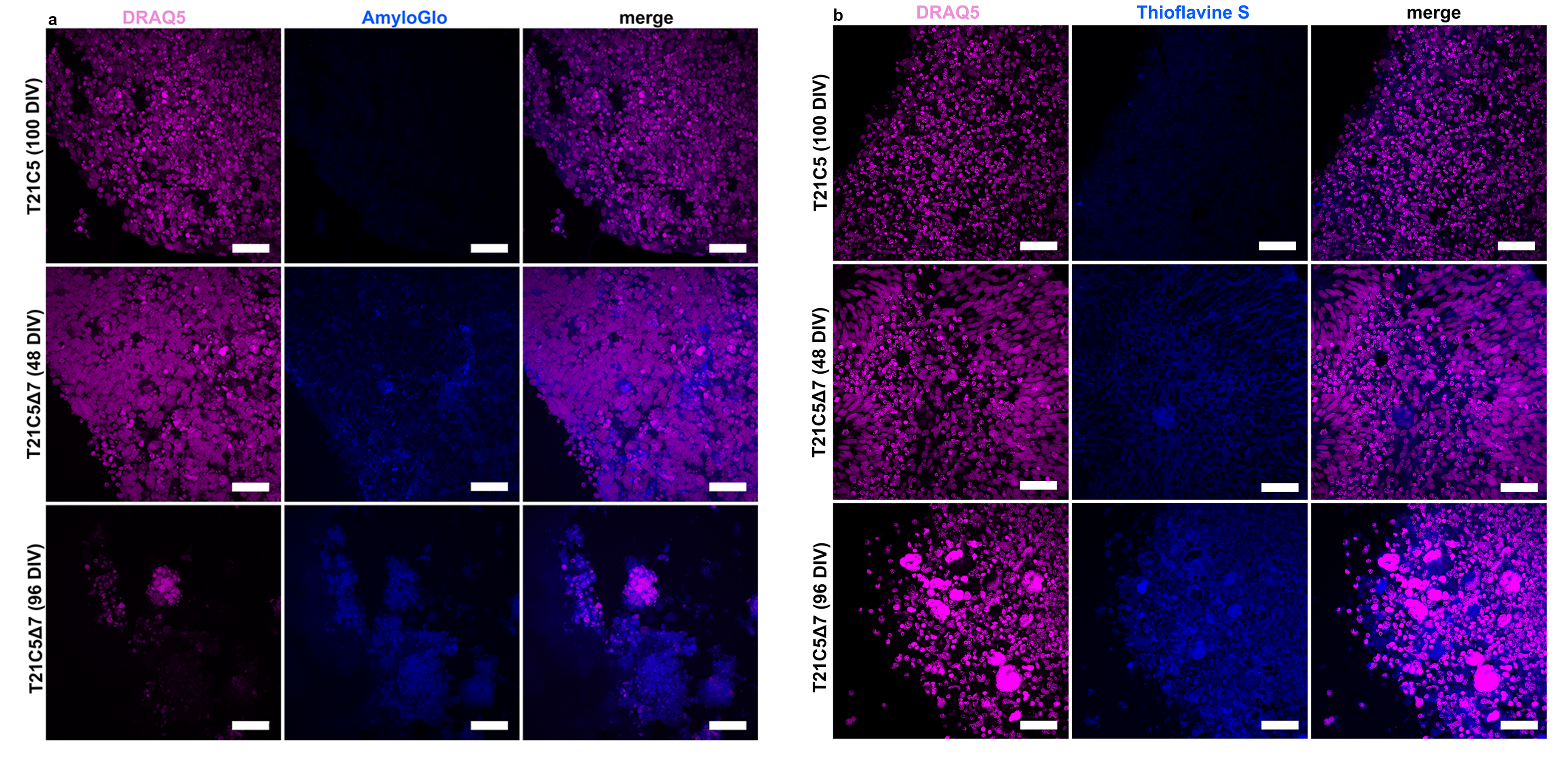

Supplement: Supplementary file 13 — Supplementary Figure 11 [file 41380_2020_806_MOESM13_ESM.tif]

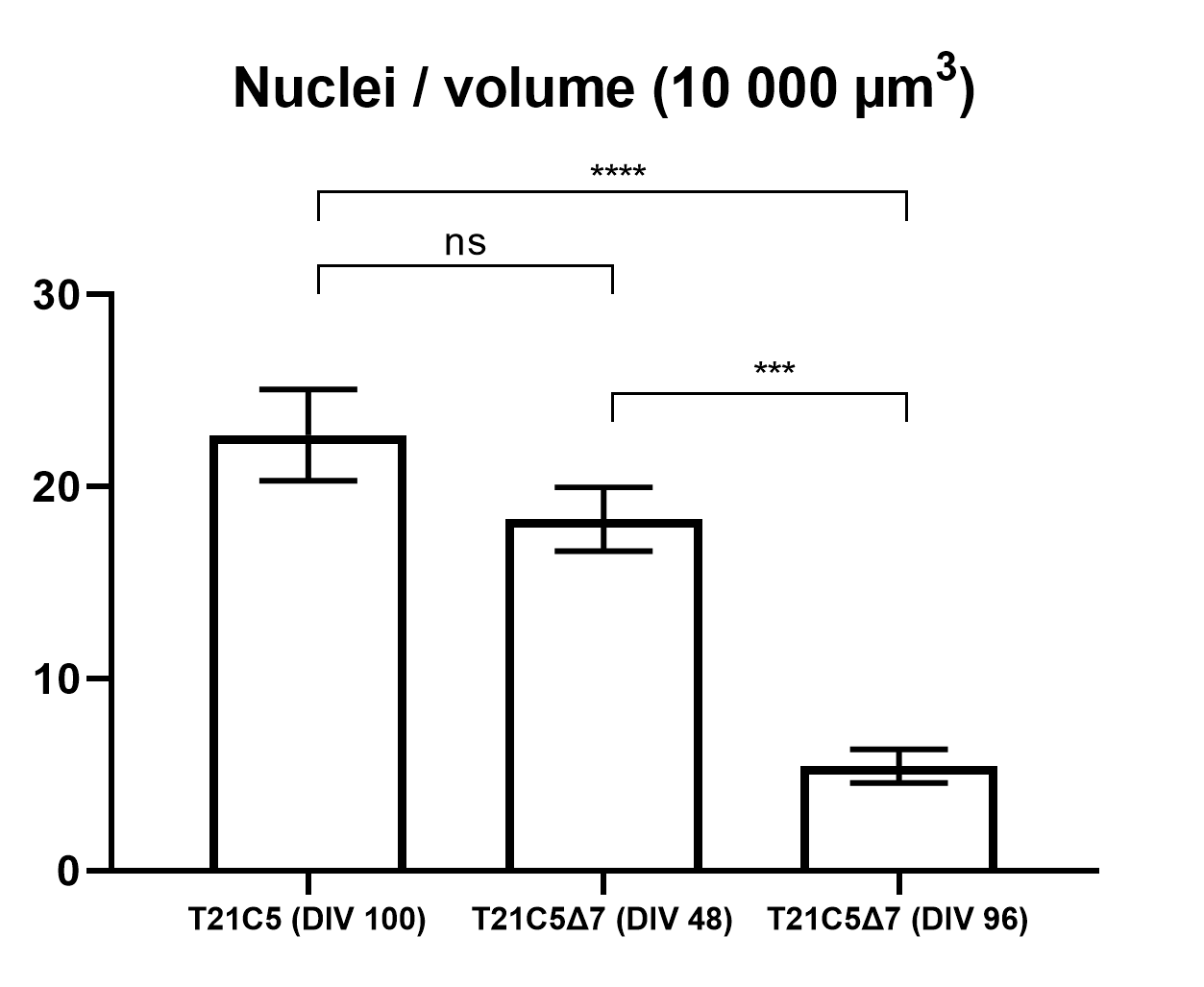

Supplement: Supplementary file 14 — Supplementary Figure 12 [file 41380_2020_806_MOESM14_ESM.tif]

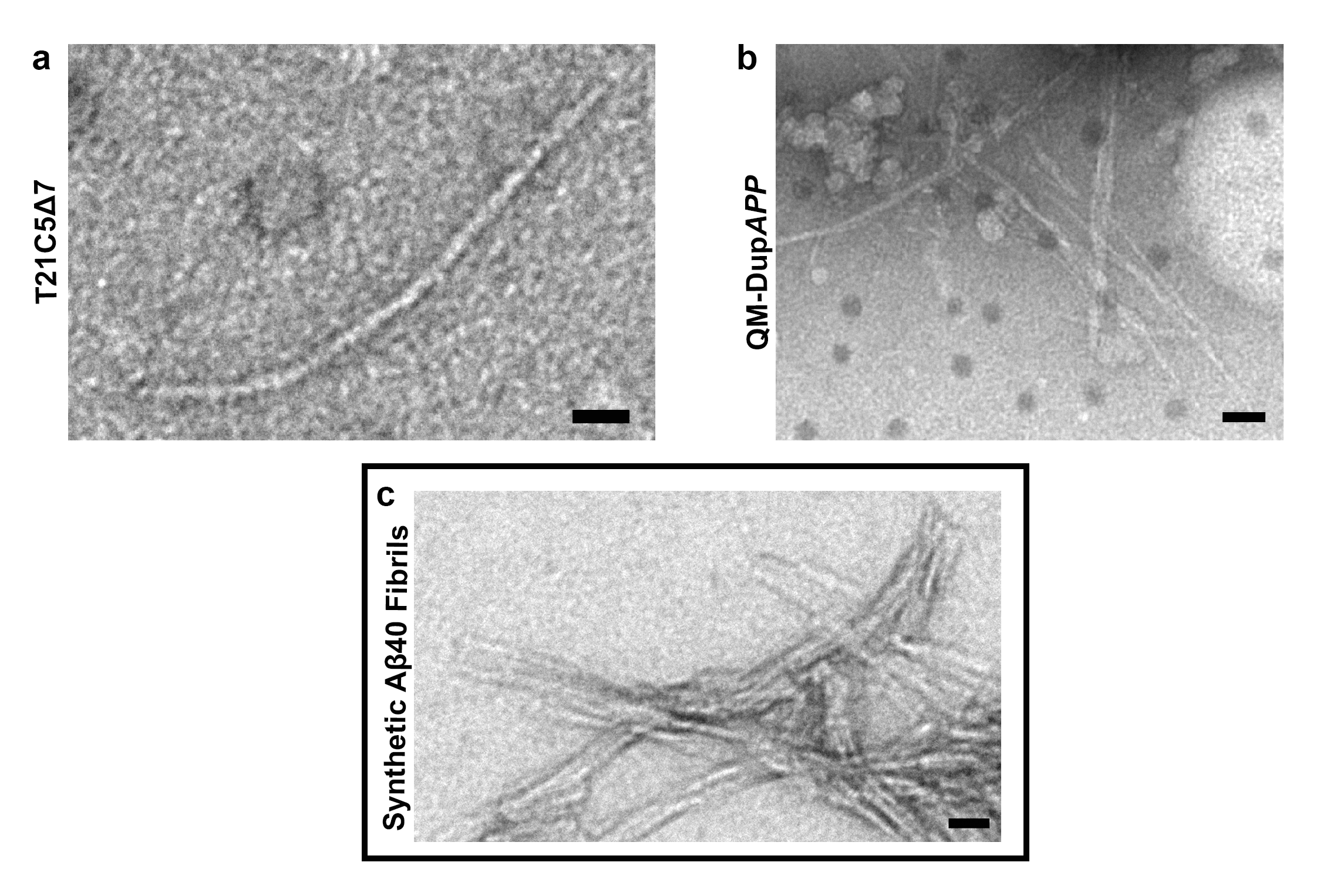

Supplement: Supplementary file 15 — Supplementary Figure 13 [file 41380_2020_806_MOESM15_ESM.tif]

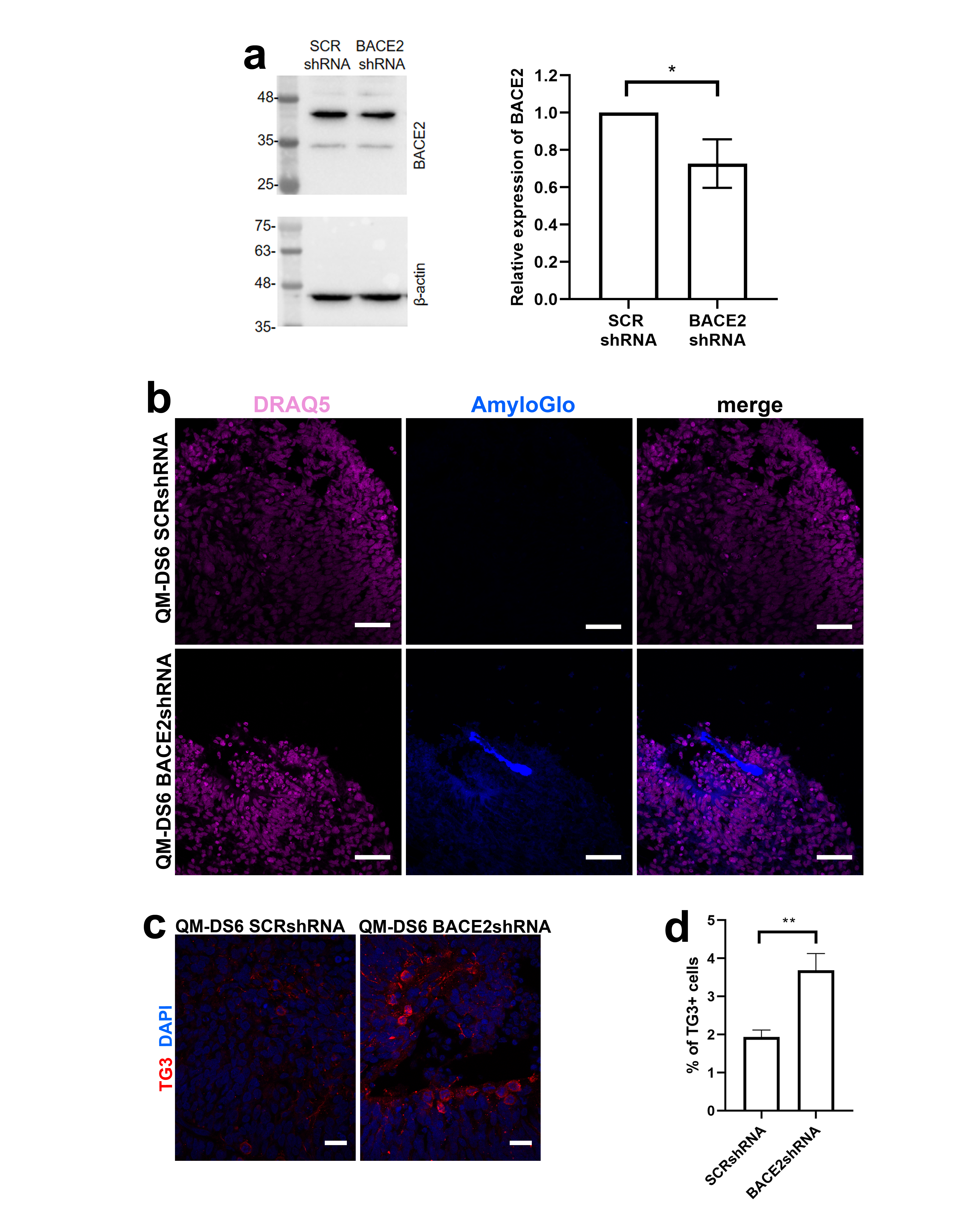

Supplement: Supplementary file 16 — Supplementary Figure 14 [file 41380_2020_806_MOESM16_ESM.tif]
