# Supplementary material for: Patient-specific Alzheimer-like pathology in trisomy 21 cerebral organoids reveals BACE2 as a gene dose-sensitive AD suppressor in human brain
Source: Mol Psychiatry. Author manuscript; Available in PMC 2022 Oct 1. (PMC8190957; doi:10.1038/s41380-020-0806-5)
Supplement: Supplementary file 1 [file NIHMS1698945-supplement-Supplementary_Information.docx]

**Supplementary Information containing Supplementary Figures, Supplementary Tables and Supplementary Data-related to some main Figures.**

Supplementary Fig. 1. Cerebral organoids express cortical neuronal layer-specific and astrocyte markers.

Supplementary Fig. 2. Comparison of the proportions of neurons and astrocytes to total cells in cerebral organoids. Isogenic D21 and T21 cerebral organoids generated mostly neurons and a small proportion of astrocytes, with no differences in the proportion of astrocytes or neurons in D21 compared to T21. Similar proportions were also detected in organoids from DupAPP, QM-DS1 and QM-DS2 iPSCs.

Supplementary Fig. 3. SNP arrays confirmed trisomy of chromosome 21 in all the iPSC lines used in this study, or in the case of QM-DupAPP, the partial duplication of a 580kbp segment of chromosome 21.

Supplementary Fig. 4. Quantitative comparison of 100DIV isogenic T21 and D21 organoid “cortical” regions by FISH and I.F.

Supplementary Fig. 5. Simplified schematic representation of APP695 from amino acid 500 to the C-terminal.

Supplementary Fig. 6. Conditioned media from isogenic D21 and T21 organoids was compared by IP-MS and ELISA.

Supplementary Fig. 7. Aβx-34 colocalises with BACE2 much more than with BACE1 in T21 cerebral organoids.

Supplementary Fig. 8. Validation and controls for immunohistochemistry.

Supplementary Fig. 9. Validation of CRISPR-edited iPSCs by SNP array and paralogous-loci-amplification-quantitative pyrosequencing, and that CRISPR-edited cerebral organoids express cortical neuronal layer-specific markers.

Supplementary Fig. 10. CRISPR/SpCas9-HF1-mediated reduction of BACE2 copy number from 3 to 2 in the T21C5 hiPSC line, reduced BACE2 protein expression to disomic levels, but does not alter the level of APP protein. 

Supplementary Fig. 11. Staining of extracellular β-amyloid deposits in organoids with two different methods.

Supplementary Fig. 12. Relative quantification of cell death in CRISPR-edited T21C5Δ7 organoids.

Supplementary Fig. 13. Electron micrographs of negatively stained filaments isolated from insoluble fraction of the AD-like pathology containing organoid lysates.

Supplementary Fig. 14. shRNA-mediated knockdown of BACE2 in QM-DS6 reduced protein expression in iPSCs and provoked AD-like pathology.

Supplementary Fig. 15. Secondary antibody alone controls for organoid immunostaining.

Supplementary Table 1. Human Brain Samples:

Supplementary Table 2. Chemicals:

Supplementary Table 3. Primary antibodies:

Supplementary Table 4. Secondary antibodies:

Supplementary Table 5. Primer sequences used for the paralogous-loci-amplification-quantitative pyrosequencing

**Supplementary Data:**

Related to Fig. 1

Related to Fig. 2 

Related to Fig. 3. and Supplementary Fig. 7

Related to Fig. 4

Related to Fig. 5

**Supplementary Table 1. Human Brain Samples:**

| **Sample** | **Region** | **Sex** | **Age** | **PMD(h)** | **Diagnosis** | **Braak** |
| --- | --- | --- | --- | --- | --- | --- |
| DS-AD1 | Hippocampus | M | 59y | <24 | DS & AD |  |
| DS-AD2 | Frontal cortex | M | 62y | 51 | DS & AD | V |
| DS-AD3 | Frontal cortex | M | 64y | 16 | DS & AD | V |
| DS-AD4 | Frontal cortex | F | 67y | 17 | DS & AD | VI |
| DS | Hippocampus | M | 28y | <24 | DS |  |
| DS | Hippocampus | M | 8m |  | DS |  |
| sAD1 | Hippocampus | M | 73y | <24 | AD | IV/V |
| sAD2 | Hippocampus | F | 83y | <48 | AD | V |
| sAD3 | Hippocampus | F | 69y |  | AD |  |
| NDC1 | Hippocampus | M | 42y | 4.5 | NDC |  |
| NDC2 | Hippocampus | F | 84y | <24 | NDC |  |
| NDC3 | Hippocampus | F | 81y | <24 | NDC |  |
| NDC4 | Hippocampus | F | 85y | <24 | NDC |  |
| NDC5 | Hippocampus | M | 78y | <24 | NDC | I/II |

**Supplementary Table 2. Chemicals:**

| **Chemical** | **Source** | **Cat.No** | **Dilution/final concentration** |
| --- | --- | --- | --- |
| AmyloGlo | Biosensis | TR-300-AG | 1:100 |
| Beta Secretase Inhibitor IV (βI-IV) | Calbiochem | 565788 | 2.5μM (organoids) 0nM-1μM (FRET) |
| Compound E (Gamma Secretase Inhibitor XXI) | Millipore | 565790 | 6nM |
| Dako Fluorescence Medium | DAKO | S3023 | N/A |
| DAPI | Sigma | D9542 | 1:8000 |
| DAPT (Gamma Secretase Inhibitor) | Merck | 565770 | 0nM-1μM |
| DMSO | Sigma | D2650 | 1:117 |
| DRAQ5 | Abcam | ab108410 | 1:1000 |
| Formic acid | Sigma | F0507 | 87% |
| Hoechst 33342 | Life Technologies | R37605 | 1:1000 |
| LY2886721 (Beta secretase inhibitor) | Selleckchem | S2156 | 0nM-0.5μM |
| Sudan black B | Abcam | ab146284 | 0.1% in 70% EtOH |
| Thioflavine S | Sigma | T1892 | 1:100 |

**Supplementary Table 3. Primary antibodies:**

| **antibody** | **clone** | **species** | **source** | **Cat.No** | **dilution** |  |  |
| --- | --- | --- | --- | --- | --- | --- | --- |
|  |  |  |  |  | Organoids | Brain | WB |
| Aβx-34 | 1B5.4 | mouse | Ref: *Cabrera E et al. 2018* | NA | 1:1000 | 1:500 |  |
| Aβx-40 | BA27 | mouse IgG2a | Wako | 014-26923 | 1:500 | 1:250 |  |
| Aβx-42 (43) | BC05 | mouse IgG1 | Wako | 010-26903 | 1:500 | 1:250 |  |
| Aβ | 4G8 | mouse IgG2b | BioLegend | 800701 | 1:200 | 1:200 |  |
| Aβ-pE3 peptide | D5N5H | rabbit IgG | Cell Signaling | #14975 | 1:200 |  |  |
| APP | 6E10 | Mouse IgG1 | Biolegend | 803001 |  |  | 1:1000 |
| BACE1 |  | rabbit IgG | Abcam | ab2077 | 1:200 | 1:200 |  |
| BACE2 |  | rabbit IgG | Abcam | ab5670 | 1:200 | 1:200 | 1:500 |
| BACE2 |  | rabbit IgG | Abcam | ab5671 | 1:200 | 1:200 | 1:500 |
| BACE2 |  | rabbit IgG | Abcam | ab8025 | 1:200 | 1:200 |  |
| Beta-Actin |  | rabbit IgG | Abcam | ab8227 |  |  | 1:10000 |
| BRN2 |  | goat IgG | Santa Cruz | SC-6029 | 1:250 |  |  |
| CTIP2 | 25B6 | rat IgG2a | Abcam | ab18465 | 1:100 |  |  |
| EEA1 | C45B10 | rabbit IgG | Cell Signaling | #3288 | 1:200 |  |  |
| Flotillin1 |  | goat IgG | Abcam | ab13493 | 1:200 |  |  |
| FOXG1 |  | rabbit IgG | Abcam | ab18259 | 1:400 |  |  |
| GFAP |  | chicken IgY | Abcam | ab4674 | - | 1:250 |  |
| GFAP | 2.2B10 | rat IgG2a | ThermoFisher Scientific | 13-0300 | 1:1000 | - |  |
| HSC70 | 1B5 | rat IgG2a | Abcam | ab19136 | 1:200 |  |  |
| LAMP1 | D2D11 | rabbit IgG | Cell Signaling | #9091 | 1:200 |  |  |
| LAMP2 | GL2A7 | rat IgG | ThermoFisher Scientific | MA1-165 | 1:100 | 1:100 |  |
| LAMP2A |  | rabbit IgG | Abcam | ab18528 | 1:200 |  |  |
| LC3A | D50G8 | rabbit IgG | Cell Signaling | #4599 | 1:400 |  |  |
| MAP2 |  | chicken IgY | Abcam | ab5392 | 1:1000 | 1:500 |  |
| Rab7 | EPR7589 | rabbit IgG | Abcam | ab137029 | 1:200 |  |  |
| Rab7 | Rab7-117 | mouse IgG2b | Abcam | ab50533 | 1:200 |  |  |
| REELIN | 142 | mouse IgG1κ | Chemicon (Merck) | mab5366 | 1:300 |  |  |
| SATB2 |  | IgG | Abcam | ab34735 | 1:200 |  |  |
| Sortilin |  | goat IgG | R&D Systems | AF3154 | 1:200 |  |  |
| Tau (3-repeat isoform RD3) | 8E6/C11 | mouse | Millipore | #05-803 | 1:500 |  | 1:1000 |
| Tau (hyperphosphorilated) | AT8 | mouse IgG | ThermoFisher Scientific | MN1020 | 1:500 |  |  |
| Tau (filamentous) | AT100 | mouse IgG | ThermoFisher Scientific | MN1060 | 1:1000 |  |  |
| Tau (conformationally altered) | TG3 | mouse IgM | From Peter Davies (via Alzforum) | NA | 1:100 |  | 1:1000 |
| TBR1 |  | rabbit IgG | Abcam | ab31490 | 1:500 |  |  |

**Supplementary Table 4. Secondary antibodies:**

| **antibody** | **conjugate** | **source** | **Cat.No** | **dilution** |
| --- | --- | --- | --- | --- |
| Donkey anti-Mouse IgG (H + L) | Alexa Fluor 488 | ThermoFisher Scientific | A-21202 | 1:1000 |
| Donkey anti-Mouse IgG (H + L) | Alexa Fluor 555 | ThermoFisher Scientific | A-31570 | 1:1000 |
| Donkey anti-Mouse IgG (H + L) | Alexa Fluor 647 | ThermoFisher Scientific | A-31571 | 1:500 |
| Donkey anti-Rabbit IgG (H + L) | Alexa Fluor 488 | ThermoFisher Scientific | A-21206 | 1:1000 |
| Donkey anti-Rabbit IgG (H + L) | Alexa Fluor 555 | ThermoFisher Scientific | A-31572 | 1:1000 |
| Donkey anti-Rabbit IgG (H + L) | Alexa Fluor 647 | ThermoFisher Scientific | A-31573 | 1:500 |
| Donkey anti-Goat IgG (H + L) | Alexa Fluor 555 | ThermoFisher Scientific | A-21432 | 1:1000 |
| Goat anti-Mouse IgG2b (H + L) | Alexa Fluor 488 | ThermoFisher Scientific | A-21141 | 1:500 |
| Goat anti-Mouse IgM (H + L) | Alexa Fluor 568 | ThermoFisher Scientific | A-21043 | 1:500 |
| Goat anti-Rat IgG (H + L) | Alexa Fluor 568 | ThermoFisher Scientific | A-11077 | 1:1000 |
| Goat anti-Chicken IgY (H + L) | Alexa Fluor 633 | ThermoFisher Scientific | A-21103 | 1:500 |
| Goat anti-Rabbit IgG (H + L) | HRP | Abcam | ab97051 | 1:10000 |
| Goat anti-Mouse IgG (H + L) | HRP | Abcam | Ab97023 | 1:10000 |
| VECTASTAIN ABC HRP Kit | HRP | Vector | PK-4002 | 1:200 |
| Double staining Kit | HRP & AP | GBI Labs | DS202A-18 | NA |

**Supplementary Table 5. Primer sequences used for the paralogous-loci-amplification-quantitative pyrosequencing**

| Gene | F primer (5’-3’) | R primer (5’-3’) | Sequencing primer (5’-3’) | Assay used (ref) |
| --- | --- | --- | --- | --- |
| GABPA | b-CTTACTGATAAGGACGCTC | CTCATAGTTCATCGTAGGCT | TCACCAACCCAAGAAA | Deutsch et al 2004 |
| ITSN | ATTTATTGCCATGTACACTT | b-GAATCTTTAAGCCTCACATAG | ACCAAGAAAGATGGTGAC | Deutsch et al 2004 |

**Supplementary Information**

**Related to Fig. 1:**

Fig. 1a: Variability between individual iPSC lines (representing individual re-programming events) was tested by ANOVA in Exp1, where all 3 independent trisomic lines of our isogenic model were used in a single experiment. No significant differences between individual lines were found in any of the calculations shown in Fig. 1, demonstrating that our peptide-ratio-readout parameter is driven by the genotype, and not re-programming artefacts or culture history of the iPSC lines (data did not fit the allowed space, available on request). 

As peptide-ratio readouts differed slightly between three independent experiments, we are showing complete data here for each experiment individually. As shown in Fig. 1a, the difference (or the absence of difference) caused by T21 in an isogenic comparison remained stable in each of 3 experiments. In Exp3, for the ratio of 1-19/amyloidogenics, the isogenic comparison of T21 v D21 showed a p=0.027 (2-tailed t-test), which dropped to p=0.0681 after ANOVA comparison with all 5 individual samples.

Also in Exp3, we further performed an analysis by genotype groups. For the AβDP/amyloidogenics ratio, the combined T21 samples (n=3) were significantly higher than D21 (ANOVA p=0.0021), and significantly higher than Dup*APP* (ANOVA p=0.0011), whereas D21 is not significantly different from Dup*APP*. The same result was obtained for the total BACE2/amyloidogenics ratio: combined T21 (n=3) v D21, ANOVA p=0.0138; combined T21 (n=3) v Dup*APP*, ANOVA p=0.0036, and D21 v Dup*APP* shows no significant difference. The comparison of α-site cleavages (1-16&1-17)/amyloidogenics never showed any significant difference irrespective of how the samples were grouped.

**Related to Fig. 2:**

Fig. 2: The FRET assay positive control was performed using recombinant human BACE2 at 37⁰C, pH=3.5 for 2h in the R&D systems assay buffer, as specified in the manufacturer’s protocol, using the R&D systems FRET control peptide (ES010). In three technical replicates the blank-subtracted raw fluorescence readings obtained were 13,836(±130 SEM). BACE2 with the new FRET peptide for the AβDP cleavage after aa34 (in the absence of any inhibitors) gave blank-subtracted readings 10,100(±59 SEM). This was taken as the 100% value for the graphs shown in Fig. 2. For comparison, BACE1 incubated with the same FRET peptide, using the manufacturer’s assay buffer for BACE1, gave the readings of 522 (±58 SEM) in the same experiment. 

**Related to Fig. 3 and Supplementary Fig. 7:**

We compared the degree of colocalisation between either BACE1 or BACE2, and Aβx-34 clearance product in organoids, along with other markers of intra-neuronal compartments: Flotillin1 (general marker of lipid rafts), Rab7 (late endosome marker), Sortilin (a major ApoE receptor linked to Aβ catabolism), and LAMP2 (one of the lysosomal membrane proteins often used to visualize lysosomes in studies of Aβ-processing). 

Both BACE1 and BACE2, as well as Aβx-34 highly colocalised with Flotillin1, suggesting that this type of Aβ degradation takes place in lipid raft containing vesicles (Fig. 3 and Supplementary Fig. 7). However, BACE1 and BACE2 differed in vesicular sub-compartment distribution: BACE1 was highly colocalised (>0.6) with each Sortilin and Rab7 and only weakly with LAMP2 (0.22), whereas BACE2 did not co-localise with Sortilin(<0.1), but colocalised moderately with Rab7 (0.31) and highly with LAMP2 (>0.5) (Supplementary Fig. 7). Interestingly, the localisation of the Aβx-34 fragment closely resembles the pattern of BACE2, and not of BACE1: (Pearson coefficient of 0.1 with each Sortilin and Rab7, and >0.5 with LAMP2), further supporting the observation of Aβx-34 (>0.5) localisation with BACE2 and less so with BACE1, in both organoids (Fig. 3 and Supplementary Fig. 7) and human brain (Fig. 4). In order to define the compartment with the highest concentration of Aβx-34 within the endo-lysosomal system more precisely, we co-stained the Aβx-34 neo-epitope-specific antibody with other markers associated with Aβ processing: LC3A (macro-autophagosome marker), EEA1 (early endosome marker) and LAMP1 (a classical lysosome marker). Surprisingly, none of these markers showed any colocalisation, demonstrating that Aβx-34 is not present in either early endosomes, macro-autophagosomes, or classical lysosomes (Fig. 3). As Aβx-34 did not colocalise with LAMP1 or LC3A, but colocalised strongly with LAMP2, we tested a colocalisation with the components of an alternative autophagy pathway that would be compatible with this pattern of colocalisations: chaperone-mediated autophagy (CMA). Unexpectedly, we detected an extremely high level of co-localization of Aβx-34 with both HSC70 (chaperone in CMA) and LAMP2A, (the isoform of LAMP2 that is the main protein controlling the levels of CMA activity) (Fig. 3). Some intra-neuronal LAMP2A+ vesicles appear to contain both HSC70 and Aβx-34 (Fig. 3). These data suggest that AβDP activity of BACE2 is linked with the CMA pathway.

**Related to Fig. 4:**

Fig. 4a-d: As immunofluorescence on brain sections is susceptible to bright and false positive autofluorescent signals from lipofuscin granules, we confirmed the colocalisation of Aβx-34 and BACE2 using non-fluorescent, chromogenic dual labelled immunohistochemistry (Supplementary Fig. 8b), where the specificity of the BACE2 antibody was further verified by pre-absorption control with the immunogenic peptide (Supplementary Fig. 8c). This method confirmed the intra-neuronal co-localization of Aβx-34 and BACE2 signals.

**Related to Fig. 5:**

The 7bp deletion causes a frameshift at aa157 of BACE2 protein sequence. This introduces a stop codon within the protease cleavage domain at aa197. The potential off-target effects of the CRISPR guide RNA used were tested using two prediction software tools: CCTop and [http://crispr.mit.edu/](http://crispr.mit.edu/). No target sequences were found with 0, 1 or 2 mismatched nucleotides. No targets, that had three or more mismatches were overlapping between the two software predictions. In CCTop, only two sites with three mismatches, and more sites with four mismatches were found. Top 10 loci from this prediction were amplified with the putative target sequence in the middle, and sequenced in the T21 wt iPSC compared to the ∆7 iPSC line. No off-target effects of the CRISPR/SpCas9-HF1 intervention were detected.
